# Exploratory Analysis of Age-Associated Somatic Mutation Patterns and Clinicopathologic Features in Endometrial Cancer

**DOI:** 10.3390/biomedicines14102350

**Published:** 2026-10-15

**Authors:** Yunhui Li, Dong Liu, Shanshan Wu, Jinghong Liang, Shiyuan Qi, Jieru Zhu, Senwei Jiang, Xiaomao Li

**Affiliations:** Department of Gynecology, The Third Affiliated Hospital of Sun Yat-sen University, No. 600 Tianhe Road, Tianhe District, Guangzhou 510630, China; liyh257@mail.sysu.edu.cn (Y.L.);

**Keywords:** endometrial cancer, age, somatic mutation, TCGA, POLE, restricted cubic spline, molecular heterogeneity

## Abstract

**Background:** Age-related heterogeneity in endometrial cancer may reflect differences in histologic composition, molecular subtype distribution, and tumor mutational burden. **Methods:** We matched TCGA clinical age data with MAF somatic mutation data and analyzed 508 patients with non-missing age and mutation information; all displayed patient-level TCGA mutation spectra and age-related comparisons used this common analytic cohort. The primary age analysis modeled age continuously using multivariable logistic regression adjusted for histologic grade and histologic type. An observed-age cutoff scan was performed only as an exploratory descriptive analysis using patient-level two-sided Fisher exact tests, followed by Benjamini–Hochberg false-discovery rate (BH-FDR) correction across all gene-cutoff comparisons. A 49-gene candidate set, defined from somatic variants with definite or potential clinical significance in a 28-patient SYSU-3 targeted-panel cohort, was evaluated in TCGA using restricted cubic spline (RCS) logistic regression. Mutation-count and POLE-exclusion sensitivity analyses were performed. **Results:** In the primary continuous-age analysis, age-related mutation odds were estimated per one-year increase with adjustment for histologic grade and histologic type. A secondary exploratory scan identified 57 years as the cutoff that maximized the number of uncorrected significant genes and separated 140 younger from 368 older patients. At this exploratory cutoff, 3308 genes were BH-FDR significant, but 3307 showed higher mutation frequency in the younger group. The younger group had a higher nonsynonymous mutation-count proxy and a higher proportion of nonsynonymous POLE variants. After excluding POLE-variant patients, only one gene remained significant in the all-gene cutoff analysis. Among the 49 SYSU-3-derived candidates, 22 showed significant linear age effects and 21 showed significant RCS overall effects; 19 genes overlapped. In SYSU-3, PTEN, PIK3CA, and ARID1A were the most frequently affected genes among variants with definite or potential clinical significance. **Conclusions:** Age was statistically associated with somatic mutation patterns in TCGA, but the broad directional signal was strongly influenced by tumor molecular composition and POLE-related hypermutation. The 57-year value should be considered an exploratory, data-driven threshold rather than a validated biological or clinical breakpoint.

## 1. Introduction

Endometrial cancer is a common malignancy of the female reproductive tract, and its clinicopathologic and molecular characteristics may differ across age groups. Age-related differences in clinicopathologic composition have been reported. In a retrospective cohort, patients younger than 45 years more often had endometrioid histology and grade 1 tumors. Patients older than 65 years more often had papillary serous histology and grade 3 tumors [1]. Age-associated genomic differences across cancers and distinct genomic landscapes in early- and late-onset endometrial cancer have been reported [2,3,4]. Molecular classification has further shown that the POLE-ultramutated, mismatch-repair-deficient, p53-abnormal, and no specific molecular profile (NSMP) groups differ in genomic complexity, histologic presentation, and clinical outcome [5,6]. In a recent Asian endometrial cancer study, patients were stratified as young (≤50 years) or old (>50 years), and the authors reported age-associated differences in genomic and immune features across the MSK-MET Asian and EC-3rd-SYSU cohorts, including higher CTNNB1 and PTEN mutation frequencies in younger patients and higher TP53, ARID1A, and MSH6 alterations in older patients, while TMB and MSI did not differ significantly [4]. These findings support age-aware genomic analysis but also illustrate that apparent age-associated patterns depend on cohort composition, assay scope, and the selected age threshold.

In endometrial cancer, molecular subtype and selected genetic alterations can improve risk assessment and inform therapeutic opportunities [7]. The present study combined a TCGA endometrial cancer cohort with a small SYSU-3 targeted-panel cohort. We prespecified continuous-age modeling as the primary analysis and used an observed-age cutoff scan only as an exploratory descriptive analysis; we also evaluated nonlinear associations, mutation burden, and the influence of POLE-variant cases. The 57-year value had no a priori biological or clinical justification; it was retained only as a data-selected descriptive marker. The purpose was to characterize age-associated signals while explicitly separating exploratory association from biological or clinical inference.

## 2. Materials and Methods

### 2.1. Data Sources and Study Populations

TCGA clinical and somatic mutation data were obtained from the Genomic Data Commons portal (https://portal.gdc.cancer.gov/). The clinical workbook contained 518 records representing 511 unique patients; three unique patients had no recorded age. The MAF file contained mutation records for 511 unique patients. After deduplication at the patient level and matching the first 12 characters of the tumor barcode, 508 unique patients had both non-missing age at initial pathologic diagnosis and mutation information and constituted the analytic cohort. The age range was 31–90 years, with 59 distinct observed ages. To prevent denominator mixing, patient-level mutation spectra and all age-related TCGA comparisons presented in the main text were recomputed in these same 508 patients.

A separate SYSU-3 cohort included 28 patients with pathologically confirmed endometrial cancer who underwent targeted-panel testing. The cohort was used to define a candidate gene set from somatic variants with definite or potential clinical significance. This terminology is used instead of equating clinical significance tiers with pathogenicity. Variant-level events were retained for event-count summaries, while the presence of at least one qualifying variant in a gene was used for patient-level mutation frequency. The intergenic (MYC, PVT1-MYC) rearrangement/fusion record was counted as an MYC-related event, resulting in 49 candidate genes and 181 variant events. The additional inference provided by SYSU-3 is limited to defining a clinically annotated, externally observed candidate universe: it reduces the exploratory candidate space from all 18,306 TCGA genes to 49 genes with observed clinically relevant variants and permits focused continuous-age modeling. It does not estimate age effects, establish the 57-year cutoff, or provide independent validation.

### 2.2. Mutation Status and Clinical Variables

For gene-level patient analyses, a patient was classified as mutated if at least one nonsynonymous somatic variant was present in the gene. Multiple variants in the same gene within one patient counted as one mutated patient. The TCGA clinical variables included age_at_initial_pathologic_diagnosis, neoplasm_histologic_grade, stage_event_clinical_stage, and histological_type. For grade comparisons, G1 and G2 were combined as G1 + G2 and compared with G3. For histologic-type comparisons, endometrioid endometrial adenocarcinoma was compared with serous endometrial adenocarcinoma; the mixed serous and endometrioid group was excluded from that comparison.

### 2.3. Statistical Analysis

Continuous variables were summarized using the mean and standard deviation or median and interquartile range, as appropriate. Categorical variables were compared using the chi-square test or Fisher exact test when expected cell counts were small. Two-sided tests were used throughout. Continuous age was the primary exposure specification; cutoff-based comparisons were secondary and descriptive. Analyses were performed using R 4.2.1, GraphPad Prism 10, Adobe Illustrator 2020, and a scripted GO/KEGG workflow. For the reconciliation, the original annotation memberships were retained while the verified background was supplied explicitly; an accompanying g:Profiler/Ensembl audit script (enrichment_verified_background.py) records the identifier-conversion procedure, database-release metadata, mapped identifier counts, and raw reproducible request. The verified enrichment universe was the 18,306 unique gene symbols in Supplementary Table S6. SYSU-3 mutation frequencies were summarized descriptively to characterize the overall external mutation spectrum; no age-group comparison, age-cutoff scan, multiplicity correction, or pathway enrichment analysis was performed in SYSU-3.

As a secondary exploratory analysis, for the age-cutoff scan, the maximum observed age of 90 years was not treated as a valid threshold because the group older than 90 years would be empty. There were 59 distinct observed ages between 31 and 90 years; removing the maximum observed age left 58 eligible observed-age cutoffs. Thus, 31–89 years denotes the minimum and maximum of the eligible observed values, not a complete sequence of every integer age, and unobserved integer ages were not added. The 89-year cutoff was retained as an extreme exploratory record; the group older than 89 years contained the three patients aged 90 years. At each cutoff, patients were divided into ≤cutoff and >cutoff groups, and each of 18,306 genes was tested with a two-sided Fisher exact test. This yielded 18,306 × 58 = 1,061,748 gene-cutoff comparisons. The exploratory cutoff was defined as the observed cutoff producing the largest number of genes with uncorrected *p* < 0.05. BH-FDR correction across all gene-cutoff tests was the primary multiplicity correction; BH-FDR within each gene across cutoffs was retained as a secondary sensitivity analysis. The selected cutoff was not interpreted as a clinical or biological threshold. Because selection was driven by the same dataset used for gene-level testing, the cutoff may be unstable under resampling or in other cohorts; independent validation or prespecified resampling analyses would be required before any clinical use.

For continuous-age analysis, age was entered as a continuous predictor in a multivariable logistic regression model for each gene, with histologic grade and histologic type as covariates. In the matched analytic cohort, grade and histologic type were complete, so no observations were removed for missing grade or type. Mixed serous and endometrioid tumors were excluded only from the endometrioid-versus-serous descriptive contrast. Effective sample sizes varied by gene because mutation status and model convergence differed across genes. The odds ratio (OR), 95% confidence interval (CI), and *p*-value corresponded to a one-year increase in age. Only models with convergence, finite coefficient estimates, finite standard errors, and an estimable *p*-value were used for statistical inference; models that did not meet these criteria were retained as descriptive records. BH-FDR was applied across the gene-level linear models.

For the 49 SYSU-3-derived candidate genes, we fitted RCS logistic regression models in the same TCGA cohort, adjusting for histologic grade and histologic type. Knots were placed at the 5th, 35th, 65th, and 95th age percentiles (44.35, 60.00, 68.00, and 83.00 years), with 57 years used as the reference age for curve interpretation. We tested both the overall age effect and the deviation from linearity. Genes with fewer than 20 mutated patients were retained descriptively but were not fitted. Overall-effect and nonlinearity *p*-values were corrected separately by BH-FDR among eligible candidate genes.

The number of nonsynonymous variants per patient was used as a mutation-count proxy. This quantity was not converted to a numerical tumor mutational burden value expressed in mutations per megabase (mut/Mb) because the available files did not provide callable genomic territory; accordingly, it was not labeled as strict tumor mutational burden. A POLE-exclusion sensitivity analysis removed patients with at least one nonsynonymous POLE variant and repeated the all-gene age-cutoff analysis using the same 58 observed cutoffs and all-test BH-FDR procedure. The available clinical file did not contain direct MSI/MMR-deficiency or TCGA four-molecular-subtype variables; these categories were therefore not inferred. To characterize the excluded POLE events, we additionally tabulated protein changes, exon/protein positions, and consequence annotations. The POLE exonuclease-domain audit used amino-acid positions 268–471; a pathogenicity flag required CLIN_SIG to contain pathogenic or likely_pathogenic. In the 508-patient analytic cohort, 79 patients had at least one nonsynonymous POLE variant; 54 had a missense alteration in positions 268–471, and the annotated pathogenic/likely pathogenic changes were P286R (21 patients), V411L (11 patients), and L424V (1 patient). The primary sensitivity analysis nevertheless remained the prespecified broad exclusion of any nonsynonymous POLE variant, because the available data did not provide a validated molecular-subtype label.

## 3. Results

### 3.1. Data-Driven Identification of the 57-Year Exploratory Cutoff

**Figure 1:**
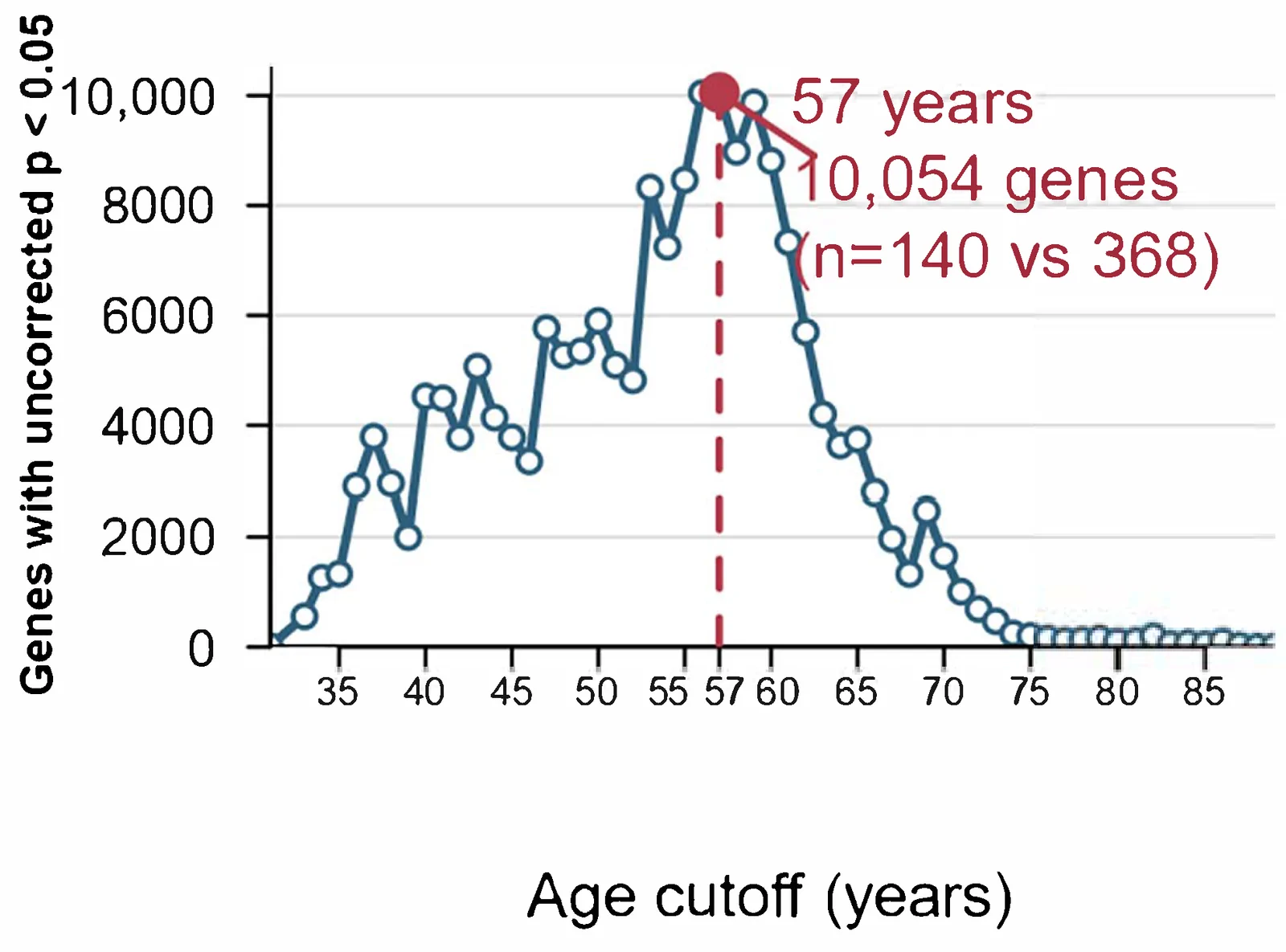
Observed-age cutoff scan for gene-level mutation-frequency differences in the TCGA cohort. Note. The *x*-axis shows each observed age cutoff and the *y*-axis shows the number of genes with an uncorrected two-sided Fisher exact *p* < 0.05. The red dashed line and marker identify the 57-year cutoff, which divided the age-associated analytic cohort into 140 younger and 368 older patients. The cutoff was selected because it maximized the number of uncorrected significant gene comparisons and should be considered exploratory rather than a validated clinical threshold. Its data-dependent selection also makes the location potentially unstable across samples and should not be interpreted as evidence for a biological discontinuity.

The continuous-age analysis was the primary model; the following cutoff scan was secondary and exploratory. Among the 508 matched TCGA patients, the observed age values ranged from 31 to 90 years, with 59 distinct observed ages. The maximum observed value of 90 years could not be used as a comparison threshold because no patient was older than 90 years; excluding that value left 58 eligible observed-age cutoffs. The eligible values ranged from 31 to 89 years, with 89 years retained as an extreme exploratory cutoff because the group older than 89 years consisted of the three patients aged 90 years. Across the 18,306 genes, 1,061,748 gene–cutoff comparisons were completed. The 57-year cutoff yielded the largest number of uncorrected *p* < 0.05 gene comparisons and was selected as the exploratory candidate cutoff. It divided the analytic cohort into a younger group of 140 patients (≤57 years) and an older group of 368 patients (>57 years). At this cutoff, 10,054 genes had uncorrected *p* < 0.05 and 3308 remained significant after BH-FDR correction across all gene-cutoff comparisons (Supplementary Table S1). This result describes the maximum within-dataset signal and does not demonstrate that 57 years is a reproducible age boundary (Figure 1).

### 3.2. Clinicopathologic Characteristics of the Age-Stratified TCGA Cohort

The younger and older groups differed significantly in histologic grade distribution (*p* = 0.0019). G3 tumors were more frequent in the older group than in the younger group (63.0% vs. 45.7%). Clinical stage distribution did not differ significantly between groups (*p* = 0.2217). Histologic type differed significantly (*p* = 3.66 × 10^−7^): endometrioid adenocarcinoma was more common in the younger group (92.1% vs. 70.1%), whereas serous adenocarcinoma was more common in the older group (4.3% vs. 25.3%) (Table 1).

### 3.3. Genomic Mutation Profile of the TCGA Cohort

**Figure 2:**
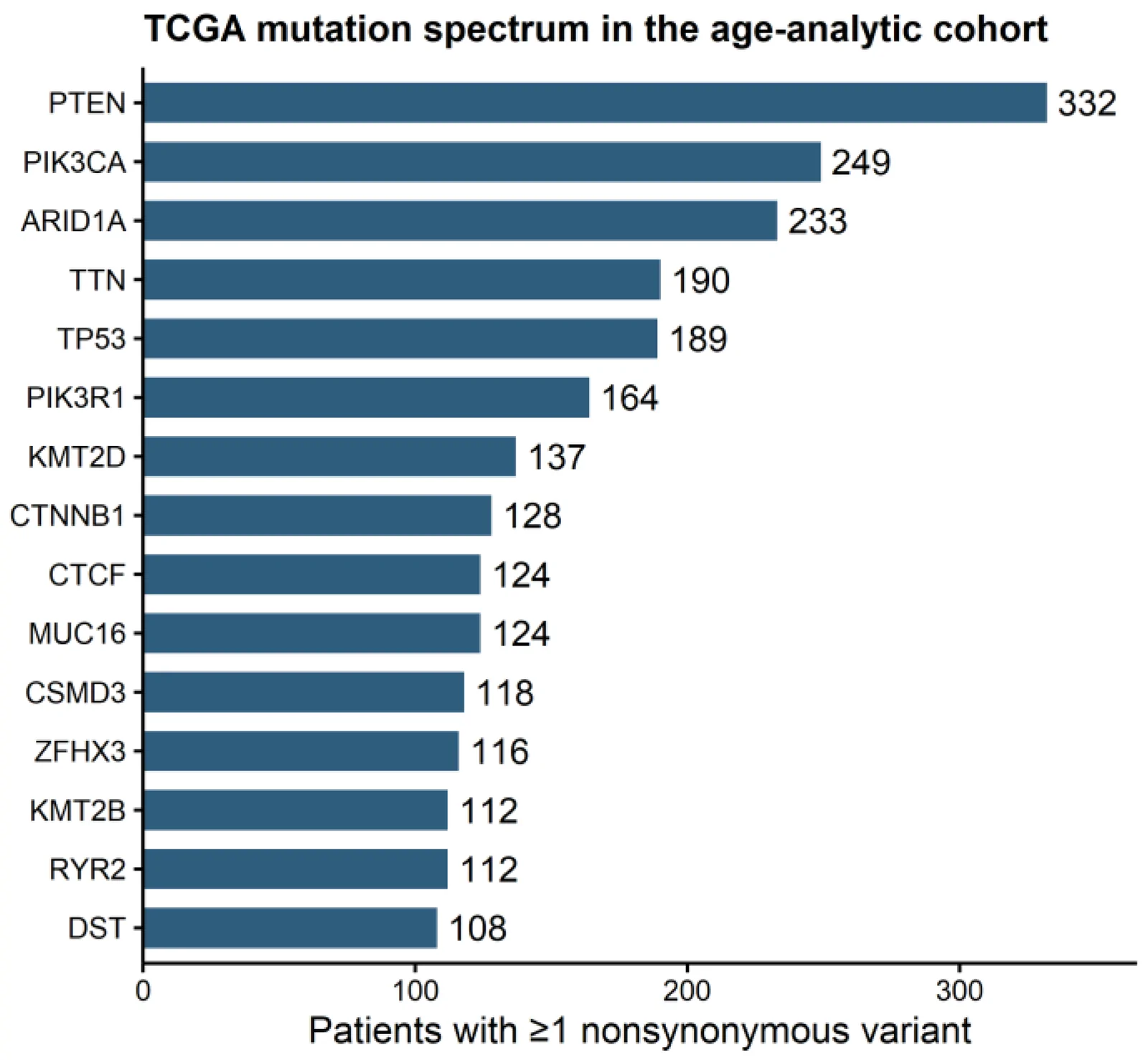
Distribution of the 15 most frequently mutated genes in the 508-patient TCGA age-analytic cohort. Note. Bars show the number of unique patients with at least one nonsynonymous mutation in each gene. The displayed denominator is the 508-patient cohort with non-missing age and matched mutation data. Counts are PTEN, 332; PIK3CA, 249; ARID1A, 233; TTN, 190; TP53, 189; PIK3R1, 164; KMT2D, 137; CTNNB1, 128; CTCF, 124; MUC16, 124; CSMD3, 118; ZFHX3, 116; KMT2B, 112; RYR2, 112; and DST, 108.

The TCGA mutation-profile description and all age-related main-text comparisons were harmonized to the 508-patient analytic cohort. The most frequently mutated genes by number of unique mutated patients were PTEN (332), PIK3CA (249), ARID1A (233), TTN (190), TP53 (189), PIK3R1 (164), KMT2D (137), and CTNNB1 (128), followed by CTCF (124), MUC16 (124), CSMD3 (118), ZFHX3 (116), KMT2B (112), RYR2 (112), and DST (108) (Figure 2).

### 3.4. Genes Associated with Age and Clinicopathologic Factors

For descriptive comparison only, using patient-level binary mutation status and two-sided Fisher exact tests at the 57-year cutoff, 3308 genes showed significant mutation-frequency differences after BH-FDR correction across all gene-cutoff tests (Supplementary Table S2). Of these, 3307 genes had higher mutation frequency in the younger group. TP53 was the only gene with higher mutation frequency in the older group (24.29% vs. 42.12%, *p* = 2.05 × 10^−4^, adjusted *p* = 0.0201). Examples with the smallest uncorrected *p*-values included CLCA2 (17.86% vs. 1.90%, *p* = 1.02 × 10^−9^, adjusted *p* = 1.36 × 10^−4^), ABCC2 (24.29% vs. 5.43%, *p* = 7.92 × 10^−9^, adjusted *p* = 4.21 × 10^−4^), and PTEN (84.29% vs. 58.15%, *p* = 1.19 × 10^−8^, adjusted *p* = 4.98 × 10^−4^). Haldane–Anscombe-corrected odds ratios for younger versus older patients were 10.64 (95% CI 4.59–24.67) for CLCA2, 5.51 (3.06–9.92) for ABCC2, and 3.79 (2.31–6.23) for PTEN; TP53 showed the opposite direction, with an odds ratio of 0.44 (0.29–0.69). These examples illustrate effect size as well as statistical ranking and direction and were not prespecified as clinical driver genes or treatment biomarkers.

In the grade comparison, G1 + G2 (*n* = 212) and G3 (*n* = 296) differed for 1016 genes after BH-FDR correction (Supplementary Table S3). Mutation frequency was higher in G3 for 1014 genes, including TP53 (8.96% vs. 57.43%, *p* = 1.72 × 10^−12^, adjusted *p* = 1.58 × 10^−8^) and PPP2R1A (5.66% vs. 21.28%, *p* = 4.51 × 10^−7^, adjusted *p* = 1.85 × 10^−3^). Two genes were more frequently mutated in G1 + G2: PTEN (85.85% vs. 50.68%, *p* = 1.57 × 10^−12^, adjusted *p* = 1.58 × 10^−8^) and CTNNB1 (39.15% vs. 15.20%, *p* = 1.21 × 10^−9^, adjusted *p* = 7.37 × 10^−6^).

**Figure 3:**
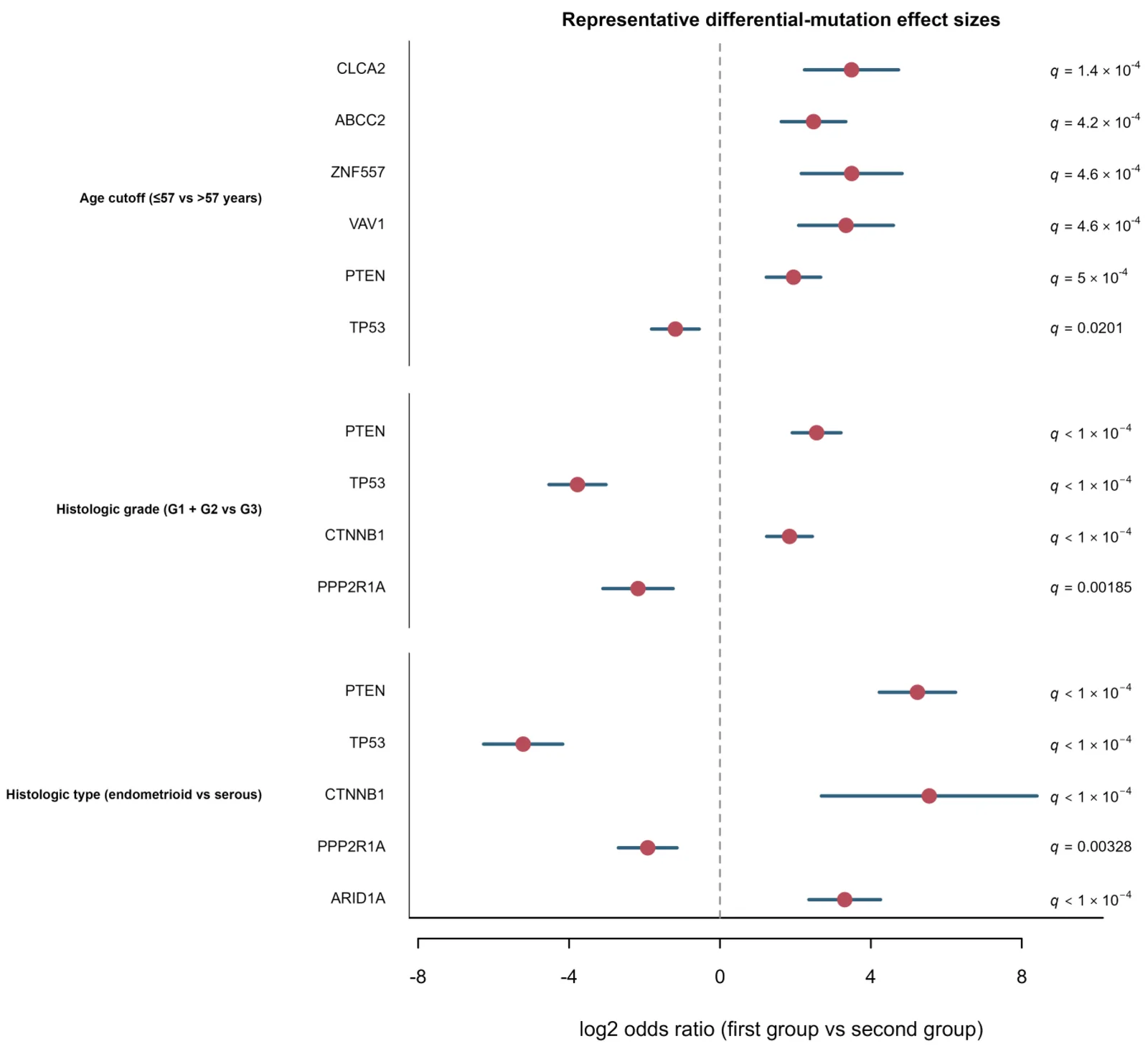
Representative effect sizes for differential mutation frequencies by age, histologic grade, and histologic type in the TCGA cohort. Note. Points show Haldane–Anscombe-corrected odds ratios (ORs) and approximate 95% confidence intervals (CIs) for the first versus second comparison group. q values are BH-FDR-adjusted *p*-values from the complete comparison tables. The displayed genes were selected from the complete comparison tables to represent the strongest ranked signals while retaining contrasting directions and nonzero cell counts; they are illustrative examples, not an independent driver-gene list. When all cells were nonzero, the uncorrected odds ratio was reported; Haldane–Anscombe continuity correction was used only when a zero cell prevented finite estimation. Age, histologic grade, and histologic type comparisons use ≤57 versus >57 years, G1 + G2 versus G3, and endometrioid versus serous adenocarcinoma, respectively.

In the histologic-type comparison, mixed histology was excluded. Endometrioid adenocarcinoma (*n* = 387) and serous adenocarcinoma (*n* = 99) differed for 145 genes after BH-FDR correction (Supplementary Table S4). Mutation frequency was higher in endometrioid tumors for 143 genes, including CTNNB1 (32.30% vs. 1.01%, *p* = 9.69 × 10^−13^, adjusted *p* = 8.31 × 10^−9^), ARID1A (55.30% vs. 11.11%, *p* = 1.38 × 10^−12^, adjusted *p* = 8.31 × 10^−9^), and PTEN (80.88% vs. 10.10%, *p* = 1.82 × 10^−12^, adjusted *p* = 8.31 × 10^−9^). TP53 (21.19% vs. 90.91%, *p* = 1.53 × 10^−12^, adjusted *p* = 8.31 × 10^−9^) and PPP2R1A (10.34% vs. 30.30%, *p* = 3.23 × 10^−6^, adjusted *p* = 3.28 × 10^−3^) were more frequently mutated in serous tumors. (Figure 3).

### 3.5. Exploratory Pre-POLE Functional Enrichment of Age-Associated Differentially Mutated Genes

**Figure 4:**
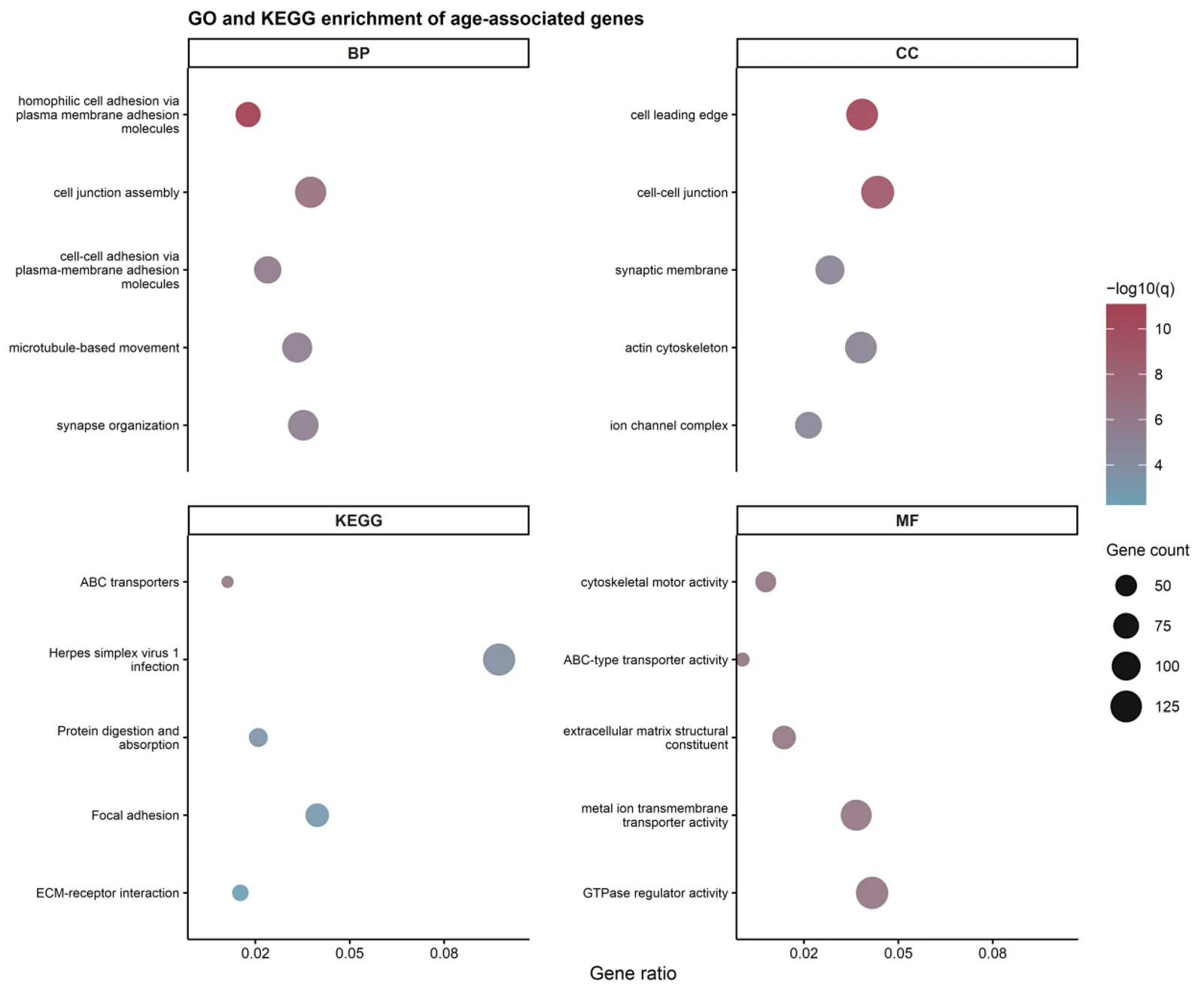
GO and KEGG enrichment of genes significant in the exploratory age-cutoff comparison in the TCGA cohort. Note. Bubble size denotes the number of genes, the *x*-axis denotes GeneRatio, and color denotes −log10(q). The five terms with the smallest BH-FDR-adjusted q values within each displayed ontology category are shown. The verified 18,306-gene TCGA universe was supplied as the background after identifier conversion; enrichment is exploratory and does not establish that the displayed pathways mediate an age effect.

To explore the potential biological functions of genes showing age-group mutation-frequency differences, we performed GO and KEGG enrichment analysis using the verified 18,306-gene TCGA analytic universe as the explicit background; the same universe was supplied to every GO and KEGG enrichment function after identifier conversion. A BH-adjusted *p* < 0.05 was considered significant. In total, 468 terms met this criterion. The five most significant terms in each of the GO biological-process, cellular-component, molecular-function, and KEGG categories are shown in Table 2. Biological processes were mainly related to homophilic cell adhesion mediated by plasma-membrane adhesion molecules, cell-junction assembly, and cell–cell adhesion. Cellular components included the cell leading edge, cell–cell junctions, synaptic membranes, and the actin cytoskeleton. Molecular functions included cytoskeletal motor activity, ABC-type transporter activity, extracellular-matrix structural constituents, metal-ion transmembrane transporter activity, and GTPase-regulator activity. KEGG pathways included ABC transporters, protein digestion and absorption, focal adhesion, and ECM–receptor interaction. These findings provide exploratory clues regarding cell adhesion, cytoskeletal organization, extracellular matrix, and transmembrane transport but do not establish that these pathways mediate age-driven tumor evolution. The post-POLE-exclusion sensitivity analysis retained only PTEN as an all-gene BH-FDR-significant gene; because a single-gene set cannot support a stable over-representation analysis, no post-exclusion pathway terms were interpreted. Accordingly, the pathway results below are presented only as pre-exclusion exploratory context and are not used to infer an age-mediated mechanism. The enrichment calculation was repeated with the verified background and is reported as exploratory context only; it does not establish that the displayed pathways mediate an age effect (Figure 4).

### 3.6. Clinicopathologic and Mutation Features of the SYSU-3 Cohort

The SYSU-3 cohort included 28 patients with pathologically confirmed endometrial cancer. Targeted-panel results and clinical-significance annotation identified 49 genes harboring somatic variants with definite or potential clinical significance and 181 cumulative variant events. By patient-level frequency, PTEN was the most frequently affected gene (23/28, 82.1%), followed by PIK3CA (21/28, 75.0%), ARID1A (18/28, 64.3%), CTNNB1 (9/28, 32.1%), KRAS (8/28, 28.6%), and FGFR2 (4/28, 14.3%). MSH6, RNF43, and TP53 were each detected in three patients (10.7%). By variant-event count, PTEN and ARID1A each accounted for 33 events (18.2%), and PIK3CA accounted for 26 events (14.4%). These three genes together accounted for 92 of 181 events (50.8%) (Table 3).

**Figure 5:**
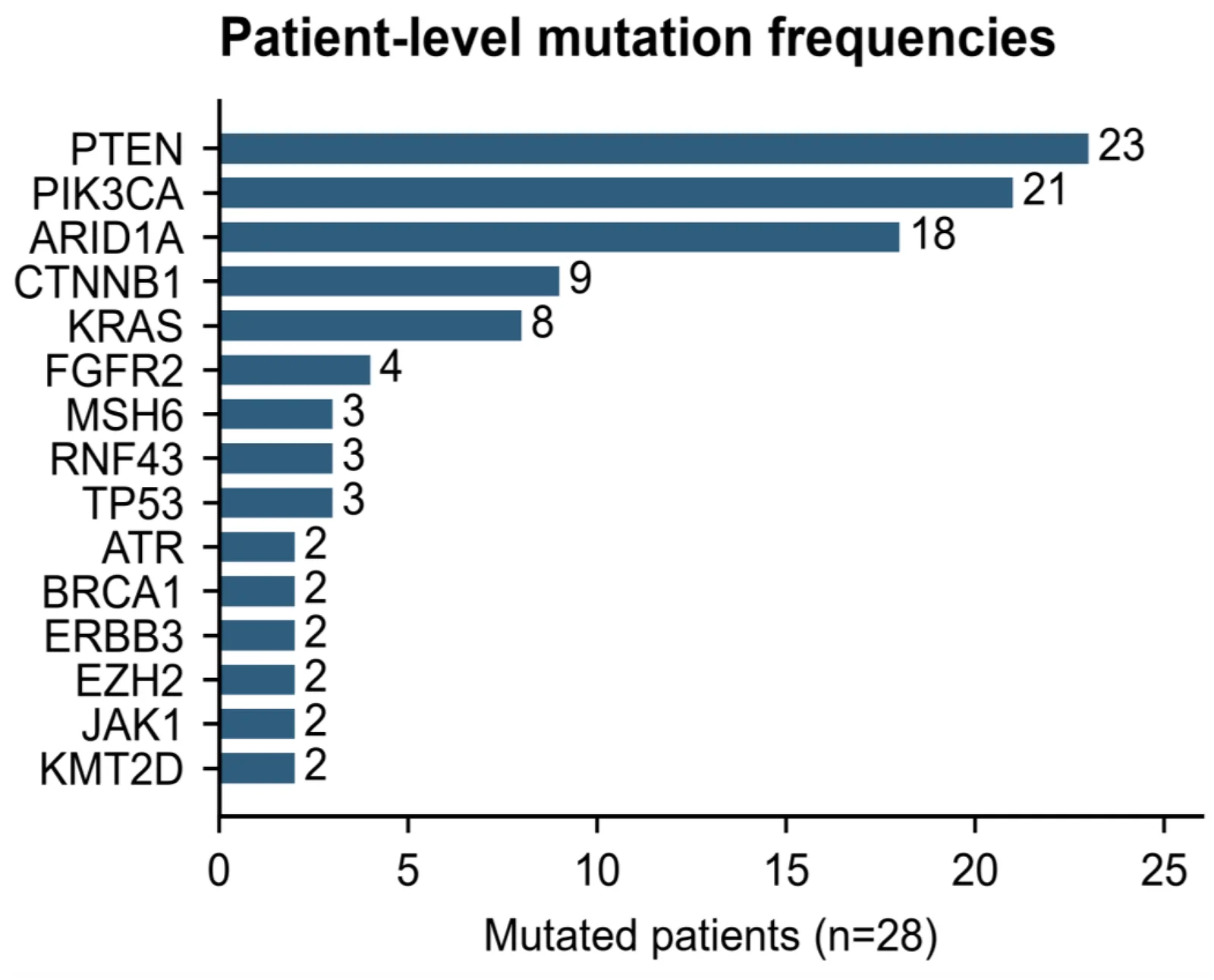
High-frequency clinically relevant somatic variants in the overall SYSU-3 external reference cohort. Note: Bars show the 15 genes with the highest numbers of patients harboring at least one qualifying somatic variant among all 28 SYSU-3 patients. Patient-level frequency counts a gene once per patient; multiple variants in the same patient–gene pair are not counted repeatedly. The intergenic (MYC, PVT1-MYC) rearrangement/fusion event was counted as an MYC-related event.

The three most frequently affected SYSU-3 genes, PTEN, PIK3CA, and ARID1A, were also among the major high-frequency genes in TCGA. This concordance is descriptive and provides external context for the core mutation spectrum; because the two cohorts differed in assay scope, variant-selection criteria, and patient composition, SYSU-3 was not used to validate the number, direction, or 57-year cutoff of TCGA age-associated genes (Figure 5). Thus, candidate selection from SYSU-3 supplies a clinically motivated filter and contextual prior, not a cross-cohort replication result (Figure 5).

### 3.7. Primary Continuous-Age Models in TCGA Using SYSU-3-Derived Candidates

In the primary continuous-age analysis within the same TCGA cohort, 22 of the 49 SYSU-3-derived candidate genes showed significant linear age effects after all-gene BH-FDR correction, and all had lower mutation odds with increasing age: MSH6, MSH2, APC, TSC1, FANCC, POLE, EZH2, MSH3, CTNNB1, CTCF, ARID1A, PTEN, BRCA1, PIK3CA, ATM, NF1, ATR, FANCM, MLH3, NFE2L2, ERBB3, and JAK1. In RCS analysis of the same 49 candidates, 43 met the threshold of at least 20 mutated patients and 40 models converged. Twenty-one genes had significant overall RCS age effects after candidate-set BH-FDR correction: MSH6, MSH2, MLH3, APC, RB1, CTCF, CTNNB1, FANCC, TSC1, EZH2, POLE, MSH3, NFE2L2, PTEN, ARID1A, ARID2, PIK3CA, ATM, BRCA1, FANCM, and NF1 (Supplementary Table S5). The complete all-gene linear results are provided in Supplementary Table S6.

**Figure 6:**
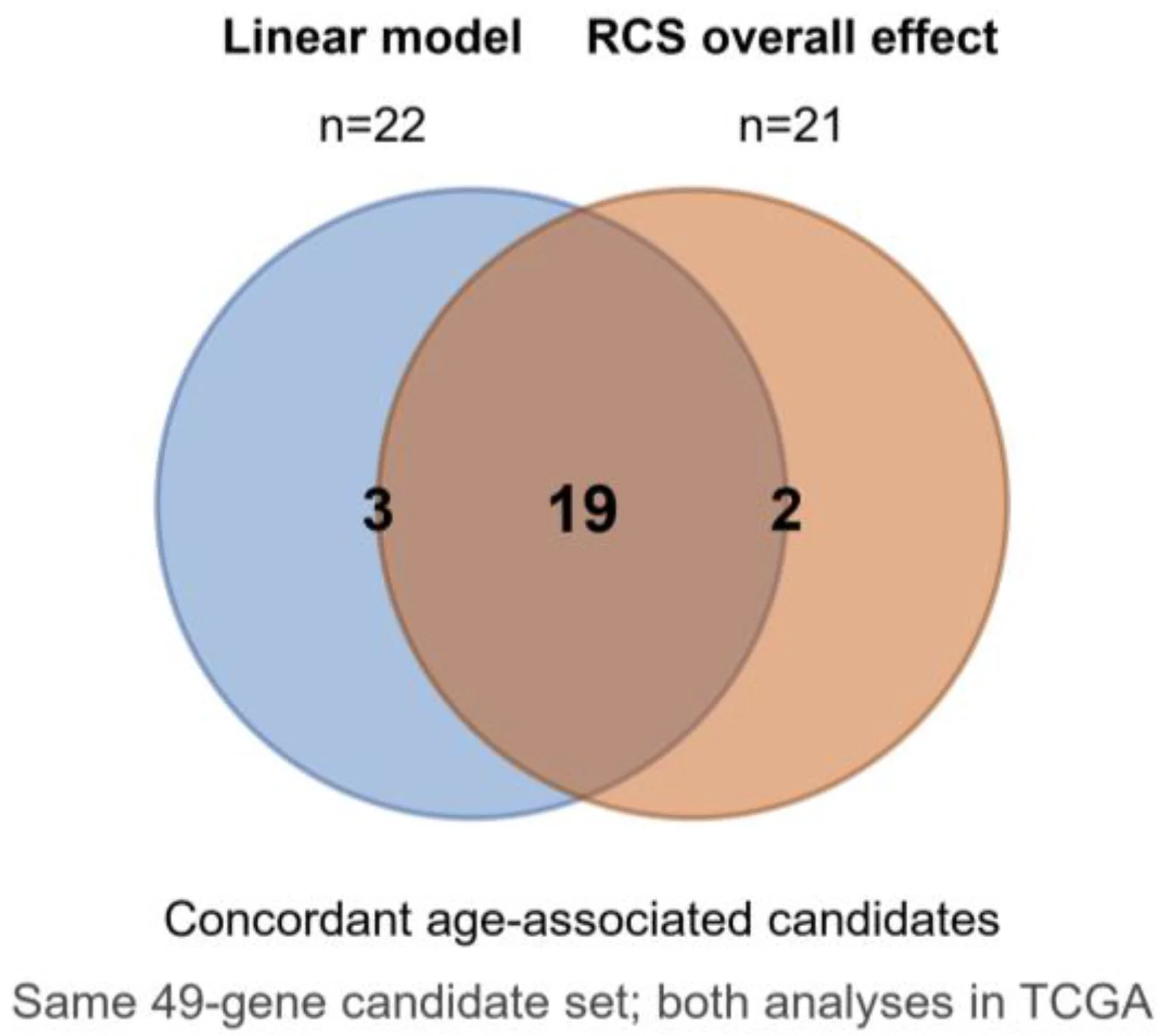
Concordance between the TCGA all-gene linear age model and RCS overall effects among SYSU-3-derived candidate genes. Note. The Venn diagram compares the same 49-gene SYSU-3-derived candidate set analyzed in TCGA. The blue circle represents the 22 genes significant in the all-gene linear model, and the orange circle represents the 21 genes significant in the RCS overall-effect model. The numbers in the non-overlapping regions indicate genes significant only in the corresponding model: 3 genes only in the linear model and 2 genes only in the RCS model. The overlapping region contains 19 genes significant in both models, such that 3 + 19 = 22 and 2 + 19 = 21. The overlap indicates model consistency rather than independent cohort validation or identification of a biological breakpoint at 57 years.

The linear and RCS overall-effect results overlapped for 19 genes: MSH6, MSH2, MLH3, APC, TSC1, FANCC, POLE, EZH2, MSH3, CTNNB1, CTCF, NFE2L2, ARID1A, PTEN, PIK3CA, ATM, NF1, BRCA1, and FANCM (Figure 6). These genes retained statistical significance under two continuous-age model specifications and therefore represent model-consistent age-associated candidates. They are not genes specifically associated with the 57-year cutoff, nor do they constitute independent validation by SYSU-3. Their RCS overall-effect estimates ranked by candidate-set BH-FDR are shown in Table 4.

### 3.8. Mutation-Count and POLE-Exclusion Sensitivity Analyses

At the 57-year exploratory cutoff, the younger group had a higher patient-level nonsynonymous mutation-count proxy than the older group (median 273.0 vs. 61.5, *p* = 0.0348). The proportion of patients with a nonsynonymous POLE variant was also higher in the younger group (27.14% vs. 11.14%, *p* = 2.82 × 10^−5^). After excluding 79 patients with a nonsynonymous POLE variant, only one gene remained BH-FDR-significant in the all-gene cutoff analysis. PTEN remained more frequent in the younger group, whereas TP53 remained more frequent in the older group, although TP53 no longer met the all-test BH-FDR threshold (Table 5); the excluded set comprised 142 MAF records in 79 analytic patients. A post hoc protein-level audit identified P286R, V411L, and L424V as the pathogenic/likely pathogenic exonuclease-domain changes under the criteria described in Methods, while other POLE variants were retained in the broad sensitivity definition.

## 4. Discussion

The primary inferential analysis modeled age continuously; this study also examined age-associated clinicopathologic and somatic mutation patterns in endometrial cancer and used an observed-age scan to identify 57 years as an exploratory candidate cutoff in TCGA. Four findings merit emphasis. First, the 57-year split produced extensive patient-level mutation-frequency differences, but the direction was highly asymmetric: 3307 of 3308 significant genes were more frequent in the younger group. Second, the younger and older groups differed in histologic grade and histologic type, with more G3 tumors and serous adenocarcinomas in the older group. Third, the younger group had a higher nonsynonymous mutation-count proxy and a higher prevalence of nonsynonymous POLE variants, and the broad age-group signal largely disappeared after POLE exclusion. Fourth, 19 SYSU-3-derived candidate genes remained significant under both continuous-age linear and RCS overall-effect models in TCGA. Together, these findings support an exploratory statistical association between age and the somatic mutation landscape but do not establish age as an independent causal driver or 57 years as a validated biological threshold.

The directionally uniform all-gene result is biologically plausible as a composition signal but should not be interpreted literally as thousands of independent age-regulated genes. Endometrial cancer molecular subtypes differ in mutation burden and clinical phenotype. POLE-ultramutated tumors, mismatch-repair-deficient tumors, p53-abnormal tumors, and tumors without a specific molecular profile have distinct genomic and clinicopathologic characteristics [5,6,7]. In our data, the younger group had a higher nonsynonymous mutation-count proxy and a higher frequency of nonsynonymous POLE variants. Removing POLE-variant patients reduced the number of BH-FDR-significant age-cutoff genes from 3308 to one. This sensitivity analysis therefore supports the interpretation that the original broad signal was at least partly driven by POLE-related hypermutation and sample-composition differences. It does not, however, define the complete POLE-ultramutated subtype and cannot remove the possible contributions of MSI-H/MMRd or copy-number-defined molecular groups because direct subtype variables were unavailable.

Clinicopathologic composition is another central explanation for the observed signal. Older patients had more G3 tumors and serous adenocarcinomas. These differences occurred against the established molecular heterogeneity of endometrial cancer [5,6,7]. Oxidative-stress-related focal-adhesion-kinase activation has also been implicated in the aggressiveness of uterine serous carcinoma [8]. Large clinical series have shown that molecular subtype and selected alterations contribute to outcome prediction and treatment stratification [7]. These patterns mean that age, grade, histologic type, molecular subtype, and mutation burden are intertwined. Although grade and histologic type were included as covariates in the continuous-age models, residual confounding is likely because the available clinical file did not contain complete molecular-subtype information, tumor purity, copy-number state, or MSI/MMR status.

The enrichment analysis supplied exploratory functional context. Age-associated differentially mutated genes were enriched in cell adhesion, cell junction, cytoskeletal, extracellular-matrix, and transmembrane-transport modules. Cell adhesion and extracellular-matrix remodeling can influence polarity, tissue architecture, migration, and invasion, and extracellular-matrix alterations have been discussed in endometrial and cervical cancer [9]. However, mutation enrichment does not directly measure gene expression, protein activity, matrix composition, or cell behavior. Because the broad age signal was markedly attenuated after POLE exclusion, these pathway findings should be viewed as provisional and should be repeated after controlling for hypermutated tumors and molecular subtype. Multi-omic and functional validation will be required before inferring a mechanistic age-to-pathway-to-phenotype chain. Because only one gene remained significant after POLE exclusion, the post-exclusion data do not support a stable pathway-level interpretation.

The SYSU-3 cohort provided a limited but useful external descriptive reference. PTEN, PIK3CA, and ARID1A were the most frequently affected genes among variants with definite or potential clinical significance, and these genes were also prominent in the TCGA mutation spectrum. Recent comparative work in young and old Asian patients with endometrial cancer similarly highlights age-associated genomic and immunologic heterogeneity [4], while other work has linked endometrial cancer gene programs to diagnosis, prognosis, and the immune microenvironment [10]. Because SYSU-3 was a small single-center targeted-panel cohort with a different variant-selection definition from TCGA, its role was limited to contextualizing high-frequency mutation patterns rather than validating age-associated differences, the 57-year cutoff, or age-related pathways. More specifically, the SYSU-3-derived candidate set supplies a clinically motivated filter for focused modeling and helps identify genes that are observable in an external targeted-panel setting; it does not add independent evidence for an age effect because all linear and RCS models were fitted in TCGA. That study used a prespecified 50-year descriptive division, whereas the present work prespecified continuous age as the primary exposure and retained 57 years only as a data-driven exploratory cutoff. The two thresholds therefore should not be treated as biologically equivalent, and the differing TMB/MSI results should not be interpreted as contradictory because our mutation-count proxy was not expressed in mut/Mb and direct MSI/MMR measures were unavailable.

The clinical implications are therefore indirect. Established clinicopathologic factors should be integrated with molecular findings rather than considered separately. Yalcin et al. showed that lymphovascular space invasion remains an important predictor of recurrence and survival even in FIGO stage I node-negative endometrioid endometrial cancer [11], while pelvic nodal involvement, lymphovascular space invasion, deep myometrial invasion, and CA-125 were associated with para-aortic nodal involvement [12]. These observations support using molecular features as one component of a broader risk-stratification framework. Current endometrial cancer risk stratification increasingly integrates POLE, MMR, p53, and NSMP information rather than relying on age alone [5,6]. Randomized trials have shown that MMR status can modify the benefit of immune checkpoint blockade in advanced or recurrent disease: dostarlimab plus carboplatin–paclitaxel improved progression-free survival, with particularly marked benefit in dMMR/MSI-H tumors [13], and pembrolizumab plus chemotherapy improved progression-free survival in both dMMR and pMMR cohorts [14]. Age-associated mutation patterns may help identify patients for prioritized molecular testing, genetic counseling, or clinical-trial evaluation, but age should not replace molecular testing or determine treatment by itself. In younger patients, fertility goals, Lynch syndrome risk, POLE/MMR status, and endometrioid histology should be considered together. In older patients, serous or p53-abnormal features, comorbidity, and treatment tolerance require equal attention.

The age-cutoff scan and continuous-age models answer different questions. The scan searches for an exploratory threshold that maximizes the number of uncorrected between-group signals within the observed TCGA data. The continuous linear model estimates the average association between age and mutation probability across the entire age range, whereas the RCS model allows the association to depart from a straight line. Thus, 57 years is used for descriptive group comparisons, while the 19-gene overlap represents consistency between continuous-age model forms. The overlap should not be described as a set of genes specifically associated with 57 years or as an independent validation by SYSU-3. In particular, a significant RCS overall effect with a non-significant corrected nonlinearity test supports an overall age association but does not support a sudden change at 57 years. This distinction is important when interpreting flexible regression models [15]. Because the scan uses the same observations for threshold selection and gene testing, the selected location is potentially unstable and is vulnerable to selection optimism.

This study has several limitations. First, age groups differed in histologic grade and histologic type, and residual confounding by molecular subtype, tumor purity, copy-number state, and technical factors cannot be excluded. The POLE-exclusion analysis suggests that molecular composition materially influenced the broad signal, but complete MSI/MMRd and TCGA molecular subtype variables were unavailable. Second, the 57-year cutoff was selected from the same data used for testing, had no a priori biological or clinical basis, and may be unstable to resampling or cohort composition; it therefore requires independent validation and should not be presented as a confirmatory clinical threshold. Third, the 49-gene RCS analysis was restricted by event counts and model convergence, and its 19-gene overlap was derived entirely within TCGA rather than across independent cohorts. Fourth, SYSU-3 was small, single-center, and targeted-panel-based and used a different variant-selection definition from TCGA; its overall mutation spectrum and candidate-gene context were descriptive only; no age-stratified or pathway results were generated in SYSU-3. Fifth, the mutation-count proxy was not strict TMB, and the study did not evaluate survival, treatment response, or functional consequences. Future multicenter studies should prespecify the age-analysis strategy, incorporate complete molecular classification, use multivariable or stratified models to separate age from subtype and histology, and integrate sequencing, copy-number, transcriptomic, methylation, immune-microenvironment, and outcome data.

## 5. Conclusions

In the TCGA endometrial cancer cohort, age was associated with patient-level somatic mutation patterns in an exploratory analysis. The data-driven 57-year cutoff separated groups with different grade, histologic composition, mutation-count proxy, and POLE-variant prevalence, and the broad gene-level signal was substantially attenuated after POLE exclusion. Nineteen candidate genes showed consistent significance across continuous-age linear and RCS overall-effect models, but this consistency reflects model agreement within TCGA rather than independent validation or a biological breakpoint at 57 years. SYSU-3 provided a clinically annotated, small-panel external descriptive reference in which PTEN, PIK3CA, and ARID1A were the most frequently affected genes. The findings support age-aware molecular characterization while underscoring that age alone should not substitute for integrated molecular classification. The molecular observations should complement, rather than replace, established clinicopathologic factors such as LVSI, myometrial invasion, nodal status, and CA-125.

## Figures and Tables

**Table 1:** Clinical and pathological characteristics of TCGA endometrial cancer patients stratified by age.

Variable	Category	Younger Group ≤ 57 Years (*n* = 140)	Older Group > 57 Years (*n* = 368)	*p* Value
Age at initial pathologic diagnosis, years	Mean +/− SD	50.4 ± 6.7	68.8 ± 7.8	
	Median (IQR)	53 (47–55)	67 (63–74)	
	Range	31–57	58–90	
Neoplasm histologic grade	G1	34 (24.3%)	62 (16.8%)	0.0019
	G2	42 (30.0%)	74 (20.1%)	
	G3	64 (45.7%)	232 (63.0%)	
Clinical stage	Stage I	86 (61.4%)	234 (63.6%)	0.2217
	Stage II	18 (12.9%)	30 (8.2%)	
	Stage III	32 (22.9%)	82 (22.3%)	
	Stage IV	4 (2.9%)	22 (6.0%)	
Histological type	Endometrioid endometrial adenocarcinoma	129 (92.1%)	258 (70.1%)	3.66 × 10^−7^
	Mixed serous and endometrioid	5 (3.6%)	17 (4.6%)	
	Serous endometrial adenocarcinoma	6 (4.3%)	93 (25.3%)	

Note. Values are *n* (%) unless otherwise indicated. The age groups were defined using the exploratory 57-year cutoff. IQR, interquartile range; SD, standard deviation.

**Table 2:** GO and KEGG enrichment analysis of age-associated differentially mutated genes in the TCGA cohort.

Ontology	ID	Description	GeneRatio	BgRatio	*p* Value	Adjusted *p*
BP	GO:0007156	homophilic cell adhesion via plasma membrane adhesion molecules	71/3077	168/18,306	5.39 × 10^−15^	3.51 × 10^−12^
BP	GO:0034329	cell junction assembly	122/3077	420/18,306	1.99 × 10^−10^	3.25 × 10^−8^
BP	GO:0098742	cell–cell adhesion via plasma-membrane adhesion molecules	87/3077	279/18,306	1.95 × 10^−9^	1.50 × 10^−7^
BP	GO:0007018	microtubule-based movement	111/3077	395/18,306	1.00 × 10^−8^	4.09 × 10^−7^
BP	GO:0050808	synapse organization	116/3077	419/18,306	1.18 × 10^−8^	4.53 × 10^−7^
CC	GO:0031252	cell leading edge	128/3167	416/18,306	9.04 × 10^−13^	2.95 × 10^−10^
CC	GO:0005911	cell–cell junction	141/3167	497/18,306	4.92 × 10^−11^	1.07 × 10^−8^
CC	GO:0097060	synaptic membrane	101/3167	373/18,306	3.28 × 10^−7^	6.90 × 10^−6^
CC	GO:0015629	actin cytoskeleton	127/3167	499/18,306	4.60 × 10^−7^	8.33 × 10^−6^
CC	GO:0034702	ion channel complex	83/3167	294/18,306	5.74 × 10^−7^	1.01 × 10^−5^
MF	GO:0003774	cytoskeletal motor activity	46/3089	111/18,306	7.02 × 10^−10^	7.63 × 10^−8^
MF	GO:0140359	ABC-type transporter activity	27/3089	49/18,306	1.18 × 10^−9^	1.10 × 10^−7^
MF	GO:0005201	extracellular matrix structural constituent	61/3089	172/18,306	2.33 × 10^−9^	1.50 × 10^−7^
MF	GO:0046873	metal ion transmembrane transporter activity	120/3089	428/18,306	2.95 × 10^−9^	1.50 × 10^−7^
MF	GO:0030695	GTPase regulator activity	133/3089	488/18,306	2.99 × 10^−9^	1.50 × 10^−7^
KEGG	hsa02010	ABC transporters	26/1474	45/18,306	5.89 × 10^−10^	7.63 × 10^−8^
KEGG	hsa05168	Herpes simplex virus 1 infection	132/1474	495/18,306	1.47 × 10^−8^	5.31 × 10^−7^
KEGG	hsa04974	Protein digestion and absorption	38/1474	103/18,306	7.49 × 10^−7^	1.16 × 10^−5^
KEGG	hsa04510	Focal adhesion	61/1474	201/18,306	1.34 × 10^−6^	1.75 × 10^−5^
KEGG	hsa04512	ECM-receptor interaction	31/1474	88/18,306	2.23 × 10^−5^	1.32 × 10^−4^

**Table 3:** Patient-level mutation frequency and mutation-event distribution of genes harboring clinically relevant somatic variants in the SYSU-3 endometrial cancer cohort.

Gene	Number of Mutated Patients	Patient Mutation Frequency (%)	Total Mutation Count	Mutation Count Proportion (%)
PTEN	23	82.14 (95% CI 64.4–92.1)	33	18.23
PIK3CA	21	75.00 (95% CI 56.6–87.3)	26	14.36
ARID1A	18	64.29 (95% CI 45.8–79.3)	33	18.23
CTNNB1	9	32.14 (95% CI 17.9–50.7)	10	5.52
KRAS	8	28.57 (95% CI 15.3–47.1)	8	4.42
FGFR2	4	14.29 (95% CI 5.7–31.5)	5	2.76
MSH6	3	10.71 (95% CI 3.7–27.2)	3	1.66
RNF43	3	10.71 (95% CI 3.7–27.2)	3	1.66
TP53	3	10.71 (95% CI 3.7–27.2)	3	1.66
JAK1	2	7.14 (95% CI 2.0–22.6)	3	1.66
SMARCA4	2	7.14 (95% CI 2.0–22.6)	3	1.66
ATR	2	7.14 (95% CI 2.0–22.6)	2	1.10
BRCA1	2	7.14 (95% CI 2.0–22.6)	2	1.10
ERBB3	2	7.14 (95% CI 2.0–22.6)	2	1.10
EZH2	2	7.14 (95% CI 2.0–22.6)	2	1.10
KMT2D	2	7.14 (95% CI 2.0–22.6)	2	1.10
MUTYH	2	7.14 (95% CI 2.0–22.6)	2	1.10
PIK3R1	2	7.14 (95% CI 2.0–22.6)	2	1.10
POLE	2	7.14 (95% CI 2.0–22.6)	2	1.10
PPP2R1A	2	7.14 (95% CI 2.0–22.6)	2	1.10
APC	1	3.57 (95% CI 0.6–17.7)	2	1.10
ARID1B	1	3.57 (95% CI 0.6–17.7)	2	1.10
CDKN2A	1	3.57 (95% CI 0.6–17.7)	2	1.10
MYC	1	3.57 (95% CI 0.6–17.7)	2	1.10
ARID2	1	3.57 (95% CI 0.6–17.7)	1	0.55
ATM	1	3.57 (95% CI 0.6–17.7)	1	0.55
BLM	1	3.57 (95% CI 0.6–17.7)	1	0.55
CTCF	1	3.57 (95% CI 0.6–17.7)	1	0.55
DICER1	1	3.57 (95% CI 0.6–17.7)	1	0.55
ERBB2	1	3.57 (95% CI 0.6–17.7)	1	0.55
ESR1	1	3.57 (95% CI 0.6–17.7)	1	0.55
FANCC	1	3.57 (95% CI 0.6–17.7)	1	0.55
FANCE	1	3.57 (95% CI 0.6–17.7)	1	0.55
FANCM	1	3.57 (95% CI 0.6–17.7)	1	0.55
FBXW7	1	3.57 (95% CI 0.6–17.7)	1	0.55
FGFR1	1	3.57 (95% CI 0.6–17.7)	1	0.55
FH	1	3.57 (95% CI 0.6–17.7)	1	0.55
MAX	1	3.57 (95% CI 0.6–17.7)	1	0.55
MLH3	1	3.57 (95% CI 0.6–17.7)	1	0.55
MSH2	1	3.57 (95% CI 0.6–17.7)	1	0.55
MSH3	1	3.57 (95% CI 0.6–17.7)	1	0.55
NF1	1	3.57 (95% CI 0.6–17.7)	1	0.55
NFE2L2	1	3.57 (95% CI 0.6–17.7)	1	0.55
PBRM1	1	3.57 (95% CI 0.6–17.7)	1	0.55
PMS1	1	3.57 (95% CI 0.6–17.7)	1	0.55
RAD50	1	3.57 (95% CI 0.6–17.7)	1	0.55
RB1	1	3.57 (95% CI 0.6–17.7)	1	0.55
STK11	1	3.57 (95% CI 0.6–17.7)	1	0.55
TSC1	1	3.57 (95% CI 0.6–17.7)	1	0.55

Note. Mutation frequency is based on the number of patients with at least one variant in the gene; multiple variants in the same patient–gene pair count as one mutated patient. Total mutation count includes all recorded variant events. The intergenic (MYC, PVT1-MYC) rearrangement/fusion was counted as an MYC-related event. Wilson 95% confidence intervals were added to each patient-level frequency; they quantify sampling uncertainty in this 28-patient descriptive cohort.

**Table 4:** Nineteen concordant age-associated candidate genes ranked by RCS overall BH-FDR.

Gene	Mutated *n* (%)	OR per 1-Year Age Increase (95% CI)	Linear *p*	Linear BH-FDR	RCS Overall *p* (BH-FDR)	Nonlinearity *p* (BH-FDR)
MSH6	72 (14.17)	0.946 (0.922–0.969); per 10-year OR 0.574 (95% CI 0.444–0.73)	9.92 × 10^−6^	0.0022	3.95 × 10^−5^ (0.0014)	0.4150 (0.7418)
MSH2	36 (7.09)	0.936 (0.906–0.967); per 10-year OR 0.516 (95% CI 0.373–0.715)	7.21 × 10^−5^	0.0036	7.04 × 10^−5^ (0.0014)	0.0878 (0.4886)
MLH3	47 (9.25)	0.965 (0.939–0.991); per 10-year OR 0.7 (95% CI 0.533–0.914)	0.0091	0.0269	5.40 × 10^−4^ (0.0072)	0.0049 (0.0989)
APC	72 (14.17)	0.956 (0.934–0.979); per 10-year OR 0.638 (95% CI 0.505–0.809)	2.10 × 10^−4^	0.0047	7.57 × 10^−4^ (0.0076)	0.3035 (0.6926)
CTNNB1	128 (25.20)	0.969 (0.950–0.988); per 10-year OR 0.73 (95% CI 0.599–0.886)	0.0014	0.0102	0.0022 (0.0138)	0.1343 (0.5939)
FANCC	22 (4.33)	0.932 (0.896–0.970); per 10-year OR 0.494 (95% CI 0.333–0.737)	5.74 × 10^−4^	0.0069	0.0026 (0.0138)	0.4164 (0.7418)
TSC1	37 (7.28)	0.945 (0.916–0.975); per 10-year OR 0.568 (95% CI 0.416–0.776)	3.77 × 10^−4^	0.0059	0.0031 (0.0138)	0.7410 (0.9675)
CTCF	124 (24.41)	0.970 (0.951–0.988); per 10-year OR 0.737 (95% CI 0.605–0.886)	0.0017	0.0108	0.0031 (0.0138)	0.1633 (0.5939)
EZH2	42 (8.27)	0.953 (0.925–0.981); per 10-year OR 0.618 (95% CI 0.459–0.825)	0.0012	0.0096	0.0038 (0.0153)	0.2762 (0.6906)
POLE	79 (15.55)	0.962 (0.941–0.984); per 10-year OR 0.679 (95% CI 0.544–0.851)	7.76 × 10^−4^	0.0077	0.0079 (0.0252)	0.9613 (0.9675)
MSH3	55 (10.83)	0.958 (0.934–0.984); per 10-year OR 0.651 (95% CI 0.505–0.851)	0.0014	0.0101	0.0081 (0.0252)	0.5575 (0.7964)
NFE2L2	37 (7.28)	0.964 (0.936–0.994); per 10-year OR 0.693 (95% CI 0.516–0.942)	0.0183	0.0421	0.0082 (0.0252)	0.0466 (0.4438)
ARID1A	233 (45.87)	0.973 (0.957–0.990); per 10-year OR 0.761 (95% CI 0.644–0.904)	0.0024	0.0127	0.0100 (0.0273)	0.4115 (0.7418)
PTEN	332 (65.35)	0.967 (0.946–0.988); per 10-year OR 0.715 (95% CI 0.574–0.886)	0.0024	0.0127	0.0102 (0.0273)	0.4265 (0.7418)
PIK3CA	249 (49.02)	0.975 (0.959–0.991); per 10-year OR 0.776 (95% CI 0.658–0.914)	0.0026	0.0132	0.0144 (0.0339)	0.5544 (0.7964)
ATM	93 (18.31)	0.970 (0.949–0.991); per 10-year OR 0.737 (95% CI 0.592–0.914)	0.0045	0.0179	0.0174 (0.0387)	0.4000 (0.7418)
BRCA1	42 (8.27)	0.956 (0.928–0.984); per 10-year OR 0.638 (95% CI 0.474–0.851)	0.0024	0.0128	0.0191 (0.0394)	0.8066 (0.9675)
FANCM	54 (10.63)	0.966 (0.941–0.991); per 10-year OR 0.708 (95% CI 0.544–0.914)	0.0089	0.0265	0.0197 (0.0394)	0.2387 (0.6642)
NF1	68 (13.39)	0.966 (0.944–0.990); per 10-year OR 0.708 (95% CI 0.562–0.904)	0.0046	0.0181	0.0230 (0.0438)	0.5223 (0.7964)

Note. All models were fitted in TCGA and adjusted for histologic grade and histologic type. The 49-gene candidate set was defined from the SYSU-3 cohort. ORs are reported per one-year increase in age, with an additional per-10-year OR and 95% CI in the same column calculated as OR^10 and CI^10. RCS overall and nonlinearity *p*-values were corrected within the eligible candidate set.

**Table 5:** Patient-level nonsynonymous mutation-count proxy and POLE-exclusion sensitivity analysis at the 57-year exploratory cutoff.

Analysis	≤57 Years	>57 Years	Statistical Result or Interpretation
Nonsynonymous mutation count per patient, median (IQR)	273.0 (37.75–958.75)	61.5 (41.75–391.75)	*p* = 0.0348, Wilcoxon rank-sum test
Patients with a nonsynonymous POLE variant	38/140 (27.14%)	41/368 (11.14%)	*p* = 2.82 × 10^−5^, two-sided Fisher exact test
Primary all-gene scan	*n* = 140	*n* = 368	3308 BH-FDR-significant genes; 3307 were higher in the younger group and 1 was higher in the older group
After excluding patients with a nonsynonymous POLE variant	*n* = 102	*n* = 327	Only PTEN remained BH-FDR significant; PTEN direction was retained (81.37% vs. 53.82%, *p* = 4.40 × 10^−7^, q = 0.0425), while TP53 remained higher in the older group (19.61% vs. 44.04%, *p* = 7.46 × 10^−6^, q = 0.2921)

Note. The mutation-count proxy is the number of nonsynonymous variants per patient and is not strict TMB because callable genomic territory was unavailable. The sensitivity analysis excluded patients with at least one nonsynonymous POLE variant and repeated the all-gene age-cutoff analysis.

## Data Availability

The raw whole-exome sequencing and clinical data of the TCGA-UCEC cohort are publicly available from the GDC Data Portal. The targeted next-generation sequencing raw data and clinical metadata of the SYSU-3 single-center endometrial carcinoma cohort generated in this study are not publicly accessible due to hospital patient privacy restrictions. The de-identified analyzed datasets supporting the findings of this study are available from the corresponding authors upon reasonable written request after institutional ethics approval is obtained.

## Supplementary Materials

The following supporting information can be downloaded at https://www.mdpi.com/article/10.3390/biomedicines14102350/s1, Supplementary Table S1, observed-age cutoff scan and cutoff-level summary; Supplementary Table S2, all 3308 genes significant at the 57-year exploratory cutoff; Supplementary Table S3, differential mutation frequencies by histologic grade; Supplementary Table S4, differential mutation frequencies by histologic type; Supplementary Table S5, RCS analysis of the 49 SYSU-3-derived candidate genes; and Supplementary Table S6, continuous-age multivariable logistic regression across all 18,306 TCGA genes. SYSU-3 was used as an external descriptive reference for high-frequency clinically relevant mutated genes in Table 3 and Figure 5. The candidate set was a clinically motivated filter, not external validation.
